# Understanding neurodegeneration after traumatic brain injury: from mechanisms to clinical trials in dementia

**DOI:** 10.1136/jnnp-2017-317557

**Published:** 2019-09-21

**Authors:** Neil SN Graham, David J Sharp

**Affiliations:** 1 Brain Sciences, Imperial College London, London, UK; 2 UK DRI Care Research & Technology Centre, Imperial College London, London, United Kingdom; 3 The Royal British Legion Centre for Blast Injury Studies, Department of Bioengineering, Imperial College London, London, United Kingdom

**Keywords:** traumatic brain injury, dementia, cognition, acquired brain injury, image analysis

## Abstract

Traumatic brain injury (TBI) leads to increased rates of dementia, including Alzheimer’s disease. The mechanisms by which trauma can trigger neurodegeneration are increasingly understood. For example, diffuse axonal injury is implicated in disrupting microtubule function, providing the potential context for pathologies of tau and amyloid to develop. The neuropathology of post-traumatic dementias is increasingly well characterised, with recent work focusing on chronic traumatic encephalopathy (CTE). However, clinical diagnosis of post-traumatic dementia is problematic. It is often difficult to disentangle the direct effects of TBI from those produced by progressive neurodegeneration or other post-traumatic sequelae such as psychiatric impairment. CTE can only be confidently identified at postmortem and patients are often confused and anxious about the most likely cause of their post-traumatic problems. A new approach to the assessment of the long-term effects of TBI is needed. Accurate methods are available for the investigation of other neurodegenerative conditions. These should be systematically employed in TBI. MRI and positron emission tomography neuroimaging provide biomarkers of neurodegeneration which may be of particular use in the postinjury setting. Brain atrophy is a key measure of disease progression and can be used to accurately quantify neuronal loss. Fluid biomarkers such as neurofilament light can complement neuroimaging, representing sensitive potential methods to track neurodegenerative processes that develop after TBI. These biomarkers could characterise endophenotypes associated with distinct types of post-traumatic neurodegeneration. In addition, they might profitably be used in clinical trials of neuroprotective and disease-modifying treatments, improving trial design by providing precise and sensitive measures of neuronal loss.

## Introduction

Traumatic brain injury (TBI) is a leading cause of death and disability worldwide, with variable long-term outcomes in survivors. The personal and societal costs are high, with the total worldwide cost estimated to be $400 billion: 0.5% of the entire annual global output.^1 2^ ^s1^ Previously, TBI has generally been viewed as producing a static neurological insult. However, it is now clear that it can trigger progressive neurodegeneration and dementia. Cognitive impairments such as loss of memory, processing speed problems and executive dysfunction are common,^3 4^ and some survivors experience cognitive decline long after injury, in part due to the development of dementia.^5–7^ Long-term dementia risk appears to be elevated after TBI, an association which is most convincing for Alzheimer’s disease (AD) and Parkinson’s disease (PD),^5^ although there are significant challenges in interpreting the prevalence of post-traumatic dementias. The relationship between TBI and dementia has important public health implications. Assuming a 10% cumulative lifetime incidence of TBI and an increased relative risk for dementia of 1.5–3 times after injury (see ‘complexities interpreting the epidemiological evidence’ section), as much as 5%–15% of all dementia cases are attributable to TBI.^8^

The clinical diagnosis of post-traumatic dementias remains a challenge. This is compounded by differing definitions of the term, with some using ‘post-traumatic dementia’ to describe fixed postinjury cognitive deficits.^9 10^ In contrast, we prefer to reserve the term for those post-traumatic cognitive problems which arise from progressive neurodegenerative pathologies. Disentangling the direct effects of TBI from those of a slowly progressive neurodegenerative process using clinical history and examination alone is difficult, and there are no accepted clinical criteria for the diagnosis of chronic traumatic encephalopathy (CTE). However, taking a ‘neurodegenerative’ approach to investigating patients with chronic problems after TBI promises to clarify diagnostic uncertainty and assist with the evaluation of novel treatments. Here we review the evidence that TBI increases the risk of dementia, briefly describe mechanisms that may explain this and discuss methods for the evaluation of neurodegeneration in the context of TBI (box 1). A range of biomarkers are available, which have already been applied in the study of other neurodegenerative conditions. Taking a similar approach in TBI has the potential to (1) improve the diagnosis of individual patients; (2) assist in identifying the type and dose of injury sufficient to cause neurodegeneration; (3) improve clinical trials by enriching trial populations for high levels of progressive neurodegeneration; and (4) provide a sensitive measure of neuronal loss that could be used in clinical trials of disease-modifying or neuroprotective interventions.

Box 1 **Key points: dementia after traumatic brain injury**

- Traumatic brain injury (TBI) is associated with an increased risk of neurodegenerative disease including Alzheimer’s disease, Parkinson’s disease and chronic traumatic encephalopathy.
- All-cause dementia risk is increased by around 1.5 times, and it has been estimated that around 5% of all dementia cases worldwide may be attributable to TBI.
- The systematic use of neuroimaging and fluid biomarker measures of neurodegeneration will allow the definition of endophenotypes of post-traumatic dementias.
- Progressive neurodegeneration is common after TBI and can be identified using MRI and positron emission tomography (PET) imaging, as well as fluid biomarkers such as neurofilament light.
- There is increasing acceptance among regulators that biomarkers such as brain atrophy rates are valid trial endpoints in presymptomatic Alzheimer’s disease.
- There is a therapeutic opportunity to intervene after TBI before significant neurodegeneration takes place, and there are good reasons to think that treatments should be initially judged against biomarkers such as brain atrophy rather than clinical endpoints.
- Trial populations in this presymptomatic group could be enriched on the basis of biomarkers of axonal injury and neurodegeneration, including diffusion tensor imaging, blood neurofilament levels or amyloid positive PET scans.

## How common is dementia after head injury?

### All-subtype dementia

Epidemiological evidence links head injury with an increased risk of dementia (table 1). A recent meta-analysis of more than two million individuals showed ~1.6 times the risk of dementia after head injury.^5^ An early 2015 meta-analysis including 22 case–control studies and 8 cohort studies reported a relative risk of 1.18 times for dementia after TBI, but this did not reach statistical significance (95% CI 0.97 to 1.39).^s2^ Oddly, given the greater severity, those who lost consciousness after injury did not show elevated dementia risk in the most recent meta-analysis; this may reflect the relatively smaller number of participants in this subgroup. However, several very large register studies have since replicated the core findings linking head injury with dementia. The Finnish Care Register captured 0.5 million person-years and showed a dose–response relationship between TBI and neurodegenerative disease: moderate-severe TBI had 1.8 times the risk of dementia compared with mild TBI.^11^

**Table 1:** Key studies summarising the relationship of TBI and neurodegenerative disease

Study	N	Design	TBI exposure	Outcome	Conclusion
Barnes *et al* 12	357 558	Cohort study (US Veterans Health Administration). Mean follow-up 4.2 years.	Moderate-severe TBI (≥1). Mild TBI (≥1) with LOC. Mild TBI (≥1) without LOC.	Dementia.	HR 3.77 (3.63–3.91) HR 2.52 (2.29–2.76) HR 2.36 (2.10–2.66)
Fann *et al* 7	2 794 852	Cohort study (Danish National Patient Register). Mean follow-up 9.9 years.	Severe TBI (single). Mild TBI (single).	Dementia.	HR 1.35 (1.26–1.45) HR 1.17 (1.13–1.20)
Gardner *et al* 19	325 870	Cohort study (US Veterans Health Administration). Mean follow-up 4.6 years.	Moderate-severe TBI (≥1) Mild TBI (≥1)	PD.	HR 1.83 (1.61–2.07) HR 1.56 (1.35–1.80)
Schaffert *et al* 16	2133	Autopsy-confirmed cases from cohort studies (from US National Alzheimer’s Coordinating Center).	TBI with LOC (≥1).	AD (neuropathologically confirmed).	3.6 years earlier onset and diagnosis
Nordström and Nordström^6^	3 329 360	Cohort study (Swedish National Patient Register). Mean follow-up 15.3 years.	Severe TBI (single). Mild TBI (single).	Dementia.	OR 2.06 (1.95–2.19) OR 1.63 (1.57–1.70)
Li *et al* 5	2 013 197	Meta-analysis of 21 case–control and 11 cohort studies.	All severities (≥1).	Dementia. AD.	RR 1.63 (1.34–1.99) RR 1.51 (1.26–1.80)
Raj *et al* 11	40 639	Cohort study (Finnish Care Register). Median follow-up 10 years. Used mild TBI controls.	Moderate-severe TBI (≥1).	Dementia. PD. ALS.	HR 1.9 (1.6–2.2) HR 1.3 (0.9–1.9) (NS) HR 1.3 (0.5–3.2) (NS)
Watanabe and Watanabe^21^	511 016	Meta-analysis of 13 case–control and 3 cohort studies.	All severity TBI (single). All severity TBI (repeated).	ALS.	OR 1.23 (1.08–1.42) OR 1.17 (0.73–1.89) (NS)
Crane *et al* 17	7130	Multiple US cohort studies (Memory and Aging Project, Adult Changes in Thought Study and Religious Orders Study).	TBI with LOC >1 hour (single).	PD. PD neuropathology. Dementia. AD. AD neuropathology.	HR 3.56 (1.52–8.28) HR 2.64 (1.40–4.99) NS NS NS
Abner *et al* 15	649	Cohort study (Kentucky Biologically Resilient Adults in Neurological Studies, ‘BRAiNS’). Median follow-up 10.8 years.	All severities (≥1).	AD neuropathology (men). AD neuropathology (women).	OR 1.47(1.03–2.09) OR 1.18 (0.83–1.68) (NS)
Jafari *et al* 18	97 372	Meta-analysis of 19 case–control, 2 nested case–control and 1 cohort study.	Symptomatic TBI (single).	PD.	OR=1.57 (1.35–1.83)
Fleminger *et al* 13	346	Meta-analysis of 15 case–control studies.	TBI with LOC (single).	AD.	OR=1.58 (1.21–2.06)

Summary of key meta-analyses or significant individual studies providing evidence about neurodegenerative diseases after TBI. All other reported tests are significant.

AD, Alzheimer’s disease; ALS, amyotrophic lateral sclerosis; LOC, loss of consciousness; NS, not significant; PD, Parkinson’s disease; RR, relative risk; TBI, traumatic brain injury.

Single mild TBI has also been associated with an increased risk of dementia, although the evidence is less clear than for more severe injuries. A 2014 systematic review assessing the risk of dementia after mild TBI found only one study of sufficient quality to warrant inclusion and did not report an overall effect.^s3^ However, several studies have since been published with a different conclusion. For example, a recent large study showed a doubling of the risk of dementia following severe injuries, but also a 1.6 times increase after mild TBI.^6^ Risks continue to be elevated for long periods, with elevated rates reported after 14 and 30 years in two large studies.^6 7^ These findings have been replicated in a military study of almost 200 000 US veterans with TBI, where TBIs of varying severity were associated with an increased risk of subsequent dementia, although other investigators have not demonstrated an association.^12^ ^s4^

### Alzheimer’s disease

The relative risk of AD after TBI has been estimated in a large meta-analysis to be increased by about 1.5 times,^5^ similar to earlier estimates.^13 14^ A marginally higher risk was seen in patients who lost consciousness after TBI. Several subsequent smaller studies have however failed to demonstrate such an association.^s5 s6^ A limitation of many studies is the absence of postmortem confirmation of the diagnosis of AD. However, the Kentucky BRAiNS investigators (Biologically Resilient Adults in Neurological Studies) used postmortem data for 238 patients with TBI and reported higher rates of AD neuropathology in men but not in women with dementia after head injury.^15^ This mirrors a general trend towards greater post-TBI dementia risk in men in the observational studies. A further study of autopsy-confirmed AD cases reported an earlier symptom onset and dementia diagnosis of 3.6 years in patients with prior TBI.^16^

However, the neuropathology data are inconsistent. A recent study described autopsy data for ~450 patients with histories of self-reported TBI including loss of consciousness.^s7^ No relationship between a history of TBI and AD neuropathology was observed, although TAR DNA-binding protein 43 (TDP-43) pathology was more commonly seen in the head injury group. Dementia rates were no higher in the TBI group than in the control population. Another large study (n=525) showed no increased AD pathology in patients who had suffered a TBI, although Lewy body pathology was increased.^17^ Both studies relied on self-reported head injury. This may have resulted in an unusual clinical sample, as the clinical part (without neuropathology) of the Crane et al study (n=7130) also found no increase in clinically diagnosed all-type dementia or AD, but did find an increased risk of PD.

### Parkinson’s disease

PD risk is increased after single TBI.^17 18^ This may be the case for mild as well as moderate/severe TBI as PD risk was increased in a recent military study of outcomes after injuries of varying severities (n=320 000).^19^ Repeated mild TBI has historically been associated with parkinsonism in the context of sporting injuries such as in boxing, attracting labels such as ‘Punch Drunk’ or ‘Dementia Pugilistica’. The syndrome is not typical of idiopathic PD as extrapyramidal signs were frequently accompanied by prominent pyramidal, cerebellar and neuropsychiatric problems. Very few contemporary studies of PD have systematically investigated a relationship with repeated mild TBI. One recent study of ~700 Thai traditional boxers, of whom only 5 developed PD, did report increased risk but only in those with the highest number of professional fights (>100) during a career.^20^

### Amyotrophic lateral sclerosis

The risk of amyotrophic lateral sclerosis (ALS) has been reported to be modestly increased after single TBI.^21^ However, several subsequent large register studies have not replicated this finding.^s8 s9^ In relation to repeated injuries, one meta-analysis showed no increased risk of ALS.^21^ Several smaller studies have reported a connection in the context of sports-related head injuries. For example, elevated ALS rates are reported in former soccer players, where risk was proportional to the duration of participation.^s10–12^ This association has not consistently been reported in National Football League players.^s13 s14^

### Frontotemporal dementia

No recent meta-analysis describes the relationship between TBI and frontotemporal dementia (FTD). A small number of studies have assessed this outcome after single injuries and report increased HRs ranging from ~1.5 to 4.5 times depending on injury severity. There is no good evidence of a relationship between mild or repeated mild TBI and FTD.^s15–19^

### Chronic traumatic encephalopathy

Although CTE has distinctive neuropathological features, no consensus clinical diagnostic criteria currently exist. Hence, the prevalence of the condition is unknown. Neuropathological studies suggest a heterogeneous clinical phenotype, with substantial overlaps to the cognitive and psychiatric problems produced directly by TBI.^22^ Conversely, many reported cases of CTE are asymptomatic at the time of death.^22 23^ ^s20^ The lack of a distinct clinical phenotype associated with neuropathologically proven cases of CTE makes it particularly difficult to disentangle the direct effects of TBI from those due to progressive neurodegeneration with cross-sectional studies. This motivates the use of detailed longitudinal evaluation of patients at risk of developing CTE using the neurodegenerative approaches we describe below.^24^ ^s21^

### Complexities interpreting the epidemiological evidence

Although there is growing evidence of a link between more severe or repeated TBI and all-cause dementia/AD, there are complexities in interpreting many of the studies. A central issue is how dementia is defined. Neurologists typically use the term dementia to refer to progressive cognitive syndromes, assuming a progressive underlying neurodegenerative pathology. However, many diagnostic manuals including the WHO’s International Classification of Diseases (ICD) and the American Psychiatric Association’s Diagnostic and Statistical Manual of Mental Disorders accommodate both progressive or static cognitive deficits under a ‘dementia’ label.^9 10^ Further confusion arises from the use of ‘neurocognitive disorder’ synonymously in recent updates. In the context of TBI, this means that a patient left with a fixed cognitive deficit could be classified as having post-traumatic dementia. Indeed, the ICD contains a specific code for ‘dementia due to injury to the head’ (6D85), which refers to cognitive problems due to TBI which ‘must arise immediately following trauma…’ and does not require the cognitive impairment to progress.

**Figure 1:**
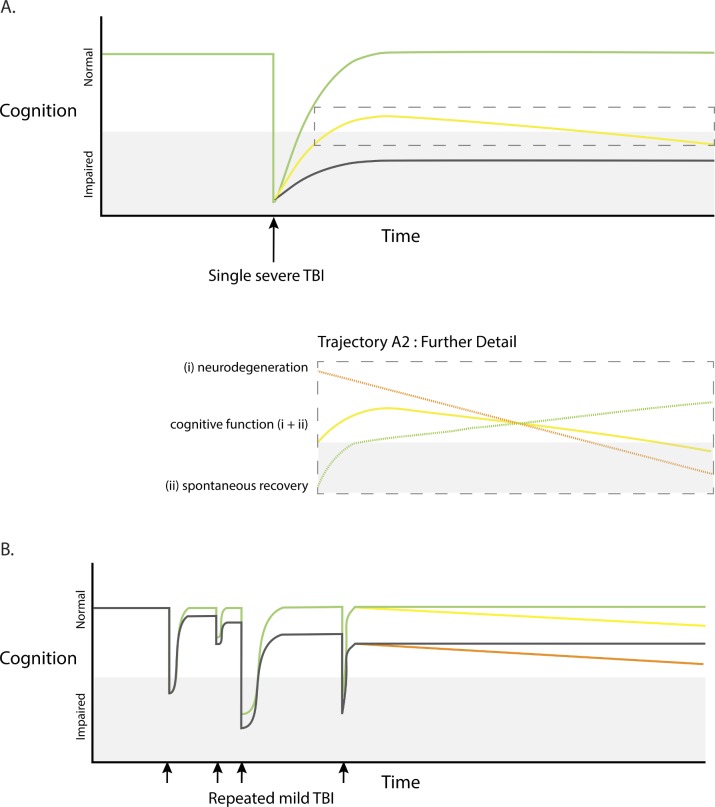
Possible cognitive trajectories after traumatic brain injury (TBI). (A) Cognitive function in relation to single severe TBI (black arrow). Marked early deterioration in cognition which may recover fully (green colour), recover partially but subsequently deteriorate (progressive neurodegeneration, yellow colour), or recover partially leaving persistent non-progressive cognitive impairment (black colour). Further detail of trajectory A2 (dashed box) illustrating that overall cognitive function (yellow colour) may be influenced by a spontaneous recovery (green colour) and neurodegeneration (orange colour). (B) Cognitive function in relation to repeated mild TBI or ‘concussions’ (small black arrows). Possible trajectories include transient impairment in cognition associated with good recoveries and no progression (green colour), or late progressive neurodegeneration (yellow colour). TBI may be followed by incomplete recovery, without late progression (grey colour) or with late progressive deterioration (orange colour).

This is an important issue, as patients often have significant cognitive impairment as a direct result of their injuries. There is also a wide range of trajectories for cognitive function after TBI, and most patients show spontaneous improvement in the initial months after injury (figure 1). This heterogeneity potentially confounds interpretation of epidemiological studies of TBI. For example, the recent Nordström study of dementia after TBI in Sweden used a generic ICD code (F03.9, ‘unspecified dementia’) which includes both static and progressive cognitive impairment. An early peak of dementia diagnosis soon after TBI was reported (HR ~3.5×), with a long tail of persistently elevated risk (1.25× 30 years after TBI). The early peak is most likely to represent the direct effects of TBI without a contribution from underlying neurodegeneration (figure 1A, green trajectory). In contrast, the persistent elevation in risk years after injury is more likely to correspond to a true increase in dementia risk due to progressive underlying neurodegeneration (figure 1A, yellow trajectory).^6^ Similar possible confounds arise when considering repeated mild TBIs, where each injury has varying spontaneous recovery as well as the possibility of triggering long-term decline related to neurodegeneration (figure 1B). In our view, the term post-traumatic dementia should be reserved for progressive neuropathology/clinical deterioration that is either suspected or confirmed, such as in CTE, AD or PD.

A further limitation of many epidemiological studies is the paucity of clinical information that is often available. This is problematic given the heterogeneity of TBI, as it makes forming judgements about the severity or associated clinical features of TBI impossible. Studies relying on self-reported TBI are particularly prone to confounds in this respect, as recall bias with respect to injury exposure is a significant problem.^25^

Genetic factors modulate neurodegenerative outcomes after TBI, and studying this relationship in large populations will inform understanding about whether an individual is likely to go on to develop dementia. The relationship between *Rep1* mutations and the development of PD is an informative example. Expansions in this *SNCA* promoter region increase alpha-synuclein expression and are associated with the development of PD. Two case–control studies (n>500) found no relationship between TBI and PD. However, patients within these studies with long expansions of *Rep1* showed increased risk of PD (ORs 3–5×).^26^ Similar relationships exist for other genetic factors: Apolipoprotein E (APOE) status is linked to adverse outcomes after TBI,^27^ ^s22^ and when combined into polygenic risk scores may predict significant amounts of variability in neurodegenerative outcomes after TBI.^s23^

## How does TBI trigger chronic neurodegeneration?

Animal models and human postmortem studies have identified pathologies of abnormal tau, amyloid beta and TDP-43 early after injuries, which may persist for months or years after TBI.^28–30^ ^s24–26^ The evidence relating early and late neuropathologies is most developed for amyloid beta and tau, where animal models show the development of pathogenic proteoforms and their subsequent evolution. The neurotoxicity of these pathogenic proteins (eg, *cis*-P-tau) contributing directly to neuronal loss seen after injury is a potential link between acute and chronic post-TBI changes.^28^ Alongside Wallerian-like degeneration, this may drive neurodegeneration and associated brain atrophy, which characterise the chronic phase of single severe TBI/CTE.^31^

**Figure 2:**
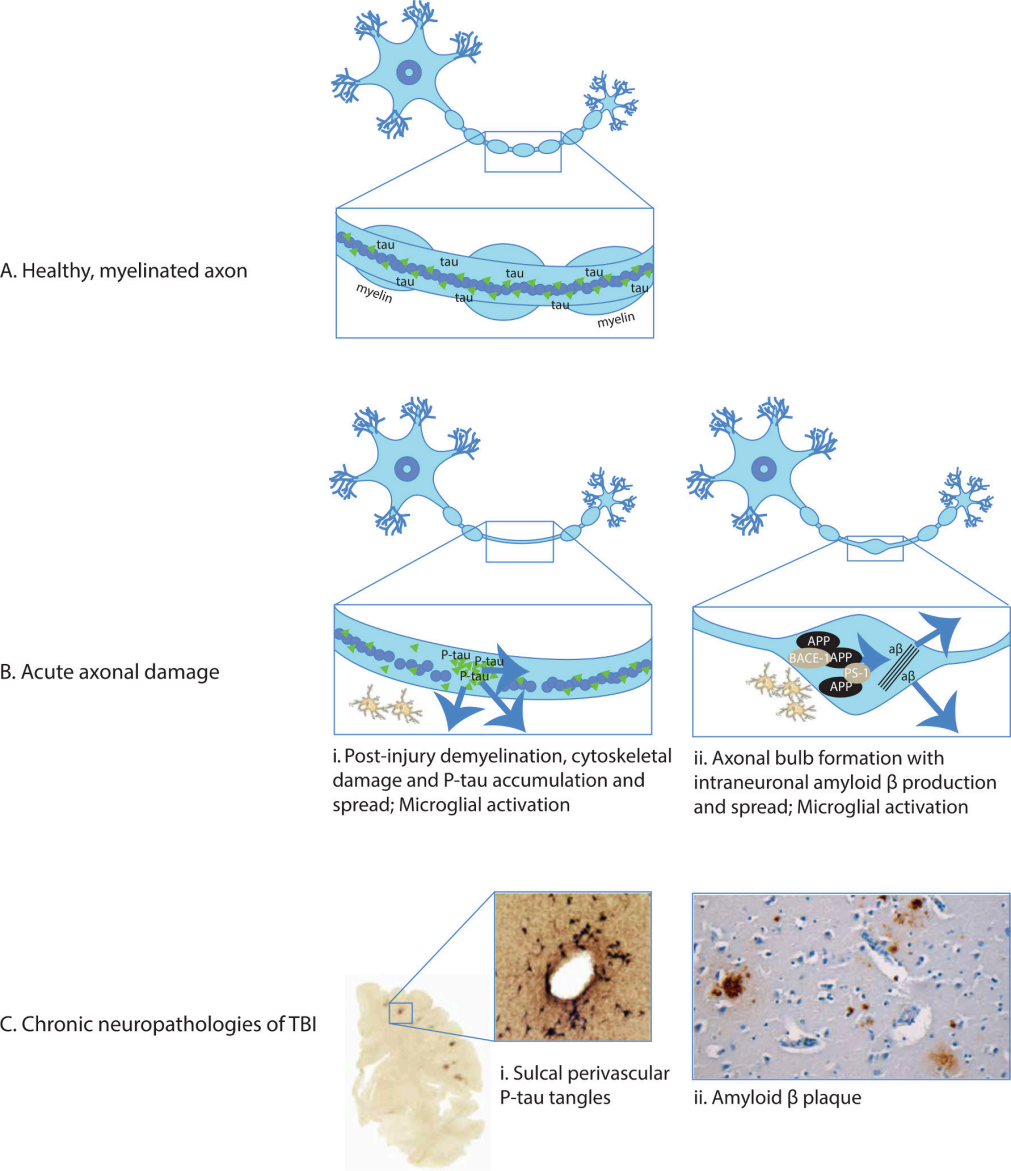
Acute neuropathologies and chronic neurodegeneration (A) Healthy, myelinated axon prior to traumatic brain injury (TBI). The box shows detail of the mid-segment of axon with central microtubules surrounded by tau with intact myelin sheath present. (B) Acute axonal damage with demyelination of the axon (panels i and ii). Tau pathology and demyelination of axon: (i) axonal injury causes cytoskeletal disruption, tau dissociation from microtubules and accumulation. Tau is aberrantly phosphorylated and may spread through extracellular, paracellular, transcellular and glymphatic mechanisms.^31^ Amyloid pathology: (ii) axonal damage causes formation of axonal bulbs/varicosities. Amyloid precursor protein (APP) accumulates with cleavage enzymes beta-site APP cleaving enzyme 1 (BACE-1) and presenilin 1 (PS-1). This produces amyloid beta which may spread to the surrounding structures following lysis of damaged neurons.^34^ Traumatic axonal damage stimulates local inflammatory response including microglial activation (panels i and ii).^31^ (C) Chronic neuropathologies. (i) Tau pathology: shearing forces during head injury localise to cortical sulcal depths causing microstructural damage, blood brain barrier disruption, axonopathy, astrogliopathy and inflammation. Sulcal perivascular localisation of P-tau neurofibrillary tangles is pathognomonic of chronic traumatic encephalopathy, visible on CP13 immunostaining.^22^ (ii) Amyloid pathology: amyloid beta plaques in a middle-aged woman who died many decades after TBI evident on immunohistochemical and thioflavine-S stains.^30^

Axonal injury is implicated as a trigger of post-traumatic neurodegenerative processes (figure 2A). Animal models and human postmortem studies of TBI show gradual Wallerian-like degeneration after injury, helping to explain the progressive white matter atrophy which characterises the chronic phase postinjury.^28^ ^s28 s29^ In addition, TBI can lead to the production of highly pathogenic species of tau and amyloid beta in the damaged axon, with further mechanistic work needed to elucidate the precise significance of axonal injury in generating progressive proteinopathies in man.^29 31 32^ ^s30^ Shearing forces applied to the cytoskeleton at the time of injury cause microstructural damage and impair axonal transport (figure 2B).^31^ Within hours of an injury, amyloid precursor protein and the cleaving enzymes beta secretase 1 and presenilin 1 accumulate in axonal varicosities. Intraneuronal amyloid beta is produced with later accumulation of extracellular plaques.^33 34^ ^s31^ Animal models of injury suggest that similar shearing forces may lead to tau dissociation from microtubules, leading to hyperphosphorylation, aggregation and aberrant processing (figure 2B).^31^ This can produce a highly pathogenic tau proteoform (*cis*-P-tau) which contributes to apoptosis, mitochondrial damage and abnormal long-term potentiation.^32^ ^s32^ It remains to be established the extent to which these mechanisms underlie the spectrum of acute and chronic pathologies seen in humans with TBI.

Post-traumatic proteinopathies have similarities to dementias, in particular AD.^35^ Tau pathology seen after TBI has a similar biochemical composition to AD but with some unique features.^s27^ CTE tau filaments have recently been characterised with cryoelectron microscopy revealing conformations quite distinct from other conditions such as AD and Pick’s disease.^36^ The localisation of tau in perivascular astrocytes at the base of brain sulci is a further distinctive feature (figure 2C).^31^ This sulcal location is predicted in computational models of the biomechanical forces at the time of injury, where strain appears to be focused at anatomical inflection points.^37^ ^s20^

TDP-43 pathologies after injury are more uncertain: cleavage is increased in animal models, generating neurotoxic fragments and promoting accumulation of ubiquitin-positive inclusions.^s16 s33^ The pathological TDP-43 observed in CTE has similarities to ALS and FTD,^s34^ although the finding is not typical of single severe injuries in man, where abnormal localisation of non-pathogenic TDP-43 is described, of uncertain significance.^s25^ Although Parkinsonian phenotypes are described after TBI, pathologies of alpha-synuclein are rarely seen, perhaps suggesting a role for other proteins such as tau (as in progressive supranuclear palsy) and substantia nigra neuronal loss.^31^

The perivascular location of tau pathology suggests that damage to the neurovascular unit may be an important causative factor in post-traumatic neurodegeneration.^22 31^ The blood brain barrier (BBB) is disrupted in the first minutes after injury, producing a complex inflammatory response in the hours and days after TBI.^38^ ^s35^^–^^38^ Microglia and astrocytes activate in response to the extravasation of proinflammatory molecules, and infiltrating monocytes contribute to the subsequent inflammatory response (figure 2B).^31^ Microglia remain activated at the site of axonal injury for many years after TBI and are associated with the long-term effects of diffuse axonal injury.^28^ The functional impact of these microglia remains uncertain, as it is unclear when they exhibit neuroinflammatory or restorative phenotypes.^28 31 39 40^ ^s38 s39^ Reduced clearance of neurodegenerative precursors may also increase late neurodegeneration, potentially caused by disruption to the normal functioning of the glymphatic system. Mouse models point to the clearance of misfolded amyloid beta and P-tau through the glymphatic system in a process dependent on the aquaporin-4 water channel located in astrocytic end-feet.^s40^ Early reactive astrogliosis following experimental TBI is associated with loss of aquaporin channels, reduced cerebrospinal fluid (CSF) flow and impaired protein clearance, suggesting a role in the subsequent accumulation of neurotoxic proteins.^s40^

A key challenge is to understand the links between the earliest stages of neurodegeneration produced at the time of injury and widespread pathological changes seen at postmortem in many cases of CTE and other types of dementia. It is proposed that CTE progresses over time in an individual to involve increasingly large parts of the brain.^22^ The staging proposal from the Boston University CTE group reflects this hypothesised progression, but it is important to note that the suggested staging is based on cross-sectional data as disease progression has not yet been characterised longitudinally in vivo. Hence, an important goal for future clinical research is to distinguish in individuals the direct effects of TBI from a truly progressive neurodegenerative process that spreads to involve neurons not necessarily affected at the time of the initial injury. An important recent observation is that TBI is capable of producing a prion-like spread of self-seeding proteinopathy. In animal models of TBI, P-tau initially present at the site of injury becomes detectable in the contralateral hemisphere 6 months after injury.^31 32 34^ In addition, local inoculation of healthy animals with contused brain homogenate induces progressive tauopathy, suggesting that brain trauma produces a transmissible self-propagating tau pathology.^41^ ^s41^

## Clinical features of neurodegeneration after TBI

We need to improve our approach to the diagnosis of post-traumatic neurodegenerative conditions. This is a difficult problem, partly because the putative clinical features of CTE (for instance, memory, behavioural and neuropsychiatric problems)^24^ and other post-traumatic dementias overlap with the direct cognitive and psychiatric effects of brain injury.^22^ For example, McKee and colleagues^22 24^ propose CTE staging based on neuropathological disease progression and highlight clinical features that are characteristic of each pathological stage. Diverse symptoms including headache, memory loss, word finding difficulty and aggression have been reported in individuals with pathologically confirmed CTE. However, these are common as a direct consequence of TBI and often persist into the chronic phase after injury. Hence, these problems are not a specific feature of post-traumatic dementia, and it is notable that no consensus clinical features of CTE have yet been established.

The neuropathological definition of CTE may be refined over time as new evidence becomes available. The current consensus NINDS/NIBIB criteria (US National Institute of Neurological Disorders and Stroke / National Institute of Biomedical Imaging and Bioengineering) are described as ‘preliminary’, reflecting the relatively small number of cases worldwide used to define the condition, risks of selection bias and difficulties due to the co-occurrence of multiple different proteinopathies in patients after TBI.^42^ ^s42^ Given the potential significance of any individual CTE diagnosis, careful distinction is required to delineate CTE pathologies from other entities such as AD pathology, primary age-related tauopathy and age-related tau astrogliopathy, which may be technically challenging.^s43 s44^ The specificity of the current consensus criteria has been criticised, with concerns raised about sensationalism in media reports of the condition, particularly given the presence of pathology in apparently healthy, asymptomatic individuals.^s45^

In our view, it is not usually possible to disentangle the direct effects of TBI from those due to neurodegenerative processes on the grounds of clinical features alone. There is unlikely to be a clinical phenotype consisting of symptoms, neurological signs and cognitive profile of sufficient specificity to allow the confident diagnosis of CTE. However, a wide range of investigations have been developed in other neurodegenerative conditions, which can usefully be applied to the study of post-traumatic neurodegeneration. These have the potential to reveal distinct and informative ‘endophenotypes’ of the underlying neurodegenerative process.^s46^ In our view the systematic use of clinical assessments in combination with multimodal biomarkers and postmortem validation will allow the development of accurate diagnostic criteria for post-traumatic dementias, as well as facilitate the measurement of disease progression and prognostication.^s47^

## How to investigate neurodegeneration after TBI

### Brain atrophy and axonal injury

Brain atrophy provides a key measure of disease progression in neurodegenerative conditions. Neuronal loss results from diverse neurodegenerative processes and produces atrophy, which can be measured using serial MRI.^43 44^ This is a sensitive although non-specific way to assess progressive neurodegeneration. It provides an integrated measure of neuronal loss seen months to years after injuries and spatial information about the pattern of this loss. A standard approach in other neurodegenerative conditions is to use repeated volumetric T1 MRI. Several refined analysis pipelines are now available, providing precise and sensitive measures how an individual’s brain changes over time.^s48 s49^ MRI is already used widely in the assessment of TBI. MRI is sensitive to contusions, haemorrhage and features associated with diffuse axonal injury such as microhaemorrhages.^1^ However, its application to the investigation of post-traumatic neurodegeneration has been surprisingly limited.

**Figure 3:**
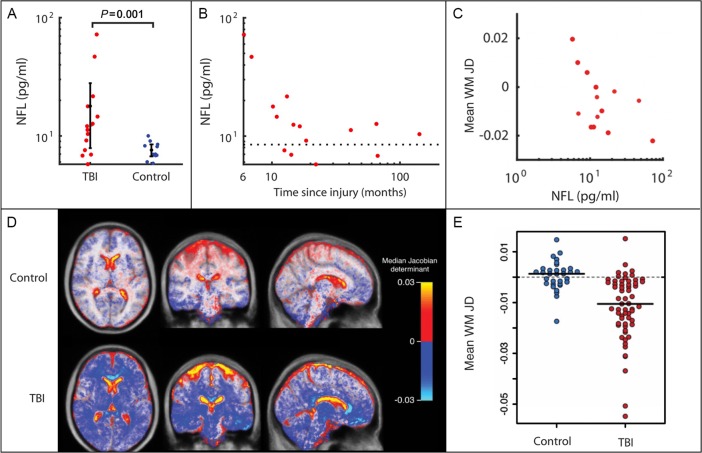
Quantifying neurodegeneration with brain atrophy and blood neurofilaments. (A) Plasma neurofilament light (NFL) levels plotted for moderate-severe traumatic brain injury (TBI) in the chronic phase and controls. Levels are significantly higher in patients with TBI than in controls.^40^ (B) NFL levels for moderate-severe TBI in the chronic phase plotted against time since injury (months).^40^ (C) Mean white matter (WM) Jacobian determinant (annualised JD rate) calculated over a 6-month scan–rescan interval in patients in the chronic phase after moderate-severe TBI, plotted against baseline plasma NFL level.^40^ (D) Spatial maps of average JD values in healthy controls and TBI patient groups. Marked progressive white matter atrophy is present after moderate-severe TBI (blue-white areas) with expansion of cerebrospinal fluid spaces (red-yellow areas) in comparison with minimal change in healthy controls.^43^ (E) Progressive atrophy of white matter following moderate-severe TBI. Scatter plot of JD rates of brain volume change in TBI compared with age-matched healthy volunteers, in white matter. A JD of 0 indicates no change in brain volume over the follow-up period.^43^

Progressive brain atrophy is very common after TBI and can be obvious when clinical scans are compared over time.^43^ Atrophy is often clear on standard neuroimaging as ventricular enlargement and cavum septum pellucidum^35^ and can be quantified using serial volumetric T1 MRI.^43^ Quantifying these changes using serial volumetric MRI shows strikingly elevated rates of atrophy, which continue for many years after a moderate-severe TBI.^43 44^ We observed a yearly loss of ~1.5% in the grey and white matter (figure 3D,E). These atrophy rates approach those seen in established AD and contrast with the absence of atrophy in healthy subjects of similar ages.^s50^ Progressive atrophy is greatest in the white matter, where widespread tracts are often affected. Higher rates are seen with greater TBI severity; lower Glasgow Coma Scale (GCS), loss of consciousness, prolonged coma and extended post-traumatic amnesia are all associated with greater atrophy.^44 45^ ^s51–54^ In our study, atrophy was substantially greater than various potentially confounding factors including ageing. High atrophy rates were seen in cortical sulci relative to gyri, possibly reflecting high strain levels which computational injury models localise to the sulci and, interestingly, corresponding to the characteristic location of CTE neuropathology in human postmortem studies.^37 42^ The extent of brain atrophy in an individual also relates to cognitive and functional outcomes after TBI,^43 44^ ^s55 s56^ with high atrophy rates associated with declining memory performance.^43^ ^s57^ Validation in clinical trials of postinjury treatments would help to establish whether atrophy is necessarily deleterious post-TBI, or if such changes are a helpful physiological response to support recovery, analogous to synaptic pruning in normal development. Although Wallerian-like degeneration of neurons is seen after injury and represents an organised response at the cellular level,^s29^ we are not aware of any convincing evidence to suggest ‘helpful’ atrophy-driven reorganisation at the global level postinjury. Our view is that the progressive and extensive loss of neurons postinjury is likely to be detrimental, akin to other neurodegenerative conditions such as AD.

Brain structure can also provide information about ageing.^45^ Variations in brain volume can be used to estimate chronological age in healthy individuals. In disease states, the discrepancy between a ‘brain age’ estimated using machine learning approaches and a patient’s chronological age can be informative. Brain age is based on volumetric information about the pattern of brain atrophy derived using T1 MRI. Machine learning is used to define the expected appearance of the brain at different ages, to which individuals are then compared. Discrepancies due to increased atrophy are reflected in an increased apparent brain age. Older than expected brain age has been reported in settings such as in patients with mild cognitive impairment and AD, and significantly, when the measure was tested in the Lothian Birth Cohort, individuals with older-appearing brains were likely to survive for a shorter duration.^46^ We have shown that moderate/severe TBI adds around 5 years to measured brain age, relative to chronological age, and that this difference predicts cognitive impairment and increases with time after injury.^47^ Hence, brains appear ‘older’ after a significant head injury, an effect that accentuates with time since injury and that correlates with post-traumatic cognitive impairments.

Diffusion MRI provides complementary information about the location and extent of diffuse axonal injury.^48^ This is relevant to post-traumatic neurodegeneration as axonal injury is linked to the production of amyloid beta and P-tau proteinopathies.^31 49^ Diffusion tensor imaging (DTI) has been widely used to investigate white matter damage produced by diffuse axonal injury (DAI), although there are several well-recognised limitations of the approach, such as difficulties assessing fibre integrity in brain regions where tracts cross one another.^s58^ Subtle abnormalities in the organisation of white matter can be detected, even when the gross scan appearances are normal. The location and severity of these changes correlate well with postinjury cognitive problems such as poor speed, executive dysfunction, memory issues and functional outcomes.^50^ Hence DTI can be used to map the presence of an important potential trigger for neurodegeneration and also provides a way to test the hypothesis that proteinopathies initiated by TBI spread in a way that is constrained by the structure of the white matter connectome.^s59^

### Fluid biomarkers of neurodegeneration

**Figure 4:**
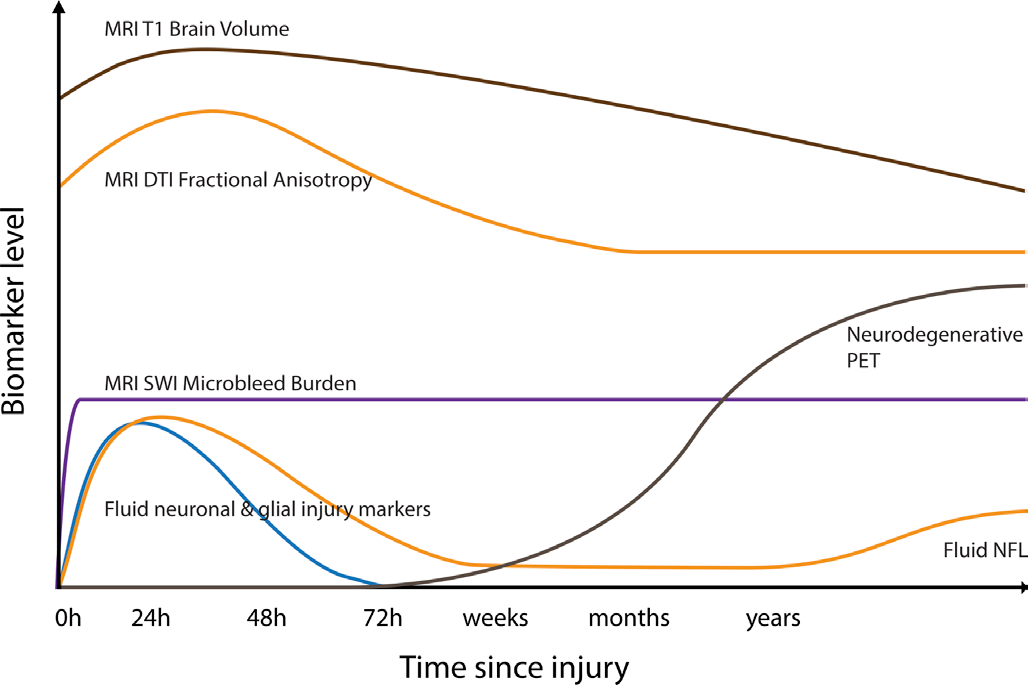
Potential longitudinal biomarker trajectories following traumatic brain injury (TBI). Hypothecated trajectories of biomarkers after moderate/severe TBI. Brain volumes measured by volumetric MRI may initially increase due to oedema before progressively reducing and continuing to decline as a result of progressive neurodegeneration after injury. Fractional anisotropy, a measure of white matter integrity derived from diffusion tensor imaging (DTI), initially increases due to acute oedema, with a subacute reduction days–weeks later reflecting axonal damage. Cerebral microbleeds, a marker of diffuse vascular injury, appear rapidly after TBI and do not resolve. They are identified most sensitively with susceptibility weighted imaging (SWI).^1^ ^s106^ Fluid neuronal and glial injury markers such as ubiquitin carboxy-terminal hydrolase L1, S100B, neuron-specific enolase, glial fibrillar acidic protein, amyloid and tau are briskly elevated after TBI. Neurofilament light levels (NFL) peak later and may be elevated in the chronic phase, correlating with progressive brain atrophy.^s107 s108^ PET, positron emission tomography.

Neuroimaging can be complemented by blood, CSF and microdialysate biomarkers of neurodegeneration (figure 4). Dramatic improvements in assay sensitivity have resulted from the transfer of standard ELISA onto the single molecule assay (Simoa) platform. This allows ultrasensitive measurement of biomarkers such as neurofilament light (NFL) and tau,^s60^ dramatically improving sensitivity for neurodegenerative conditions. NFL is a particularly promising biomarker. It is found in high concentrations within myelinated axons, and animal models of neurodegeneration show NFL to be a sensitive measure of the onset of a range of proteopathic lesions in the brain. Changes in NFL levels can be used to track disease progression and treatment response,^51^ and in humans increased levels are observed in a variety of neurodegenerative diseases, including AD and motor neuron disease.^51^ ^s61^^–^^63^ Serial NFL sampling in individuals at risk of AD predicts brain atrophy rates, cognitive impairment and disease progression.^52^ As plasma NFL levels are highly correlated with CSF NFL, blood testing of NFL is informative.^53^ ^s62^^–^^64^

NFL and tau have also been used to assess TBI, particularly in the acute setting.^54^ Blood and CSF levels increase acutely after brain injury and relate to the severity of injury.^s65 53^ Concentrations in blood and CSF rise briskly after even mild injuries, such as following a bout of contact in boxers, or head injury in ice hockey.^s66 s67^ Levels of NFL but not tau remain elevated in the chronic phase after TBI in some individuals, and levels correlate with measures of diffuse axonal injury and progressive brain atrophy (figure 3E).^40^ ^s68^ This suggests that NFL levels reflect the extent of traumatic injury, particularly to large myelinated axons. In the chronic phase, persistently increased blood NFL may indicate the presence of progressive post-traumatic neurodegeneration. If this is confirmed in larger studies, plasma NFL may prove diagnostically useful in identifying patients at risk of developing post-traumatic dementias of various types and for stratifying patient recruitment into clinical trials.

### Molecular imaging: amyloid, tau and microglial activation

**Figure 5:**
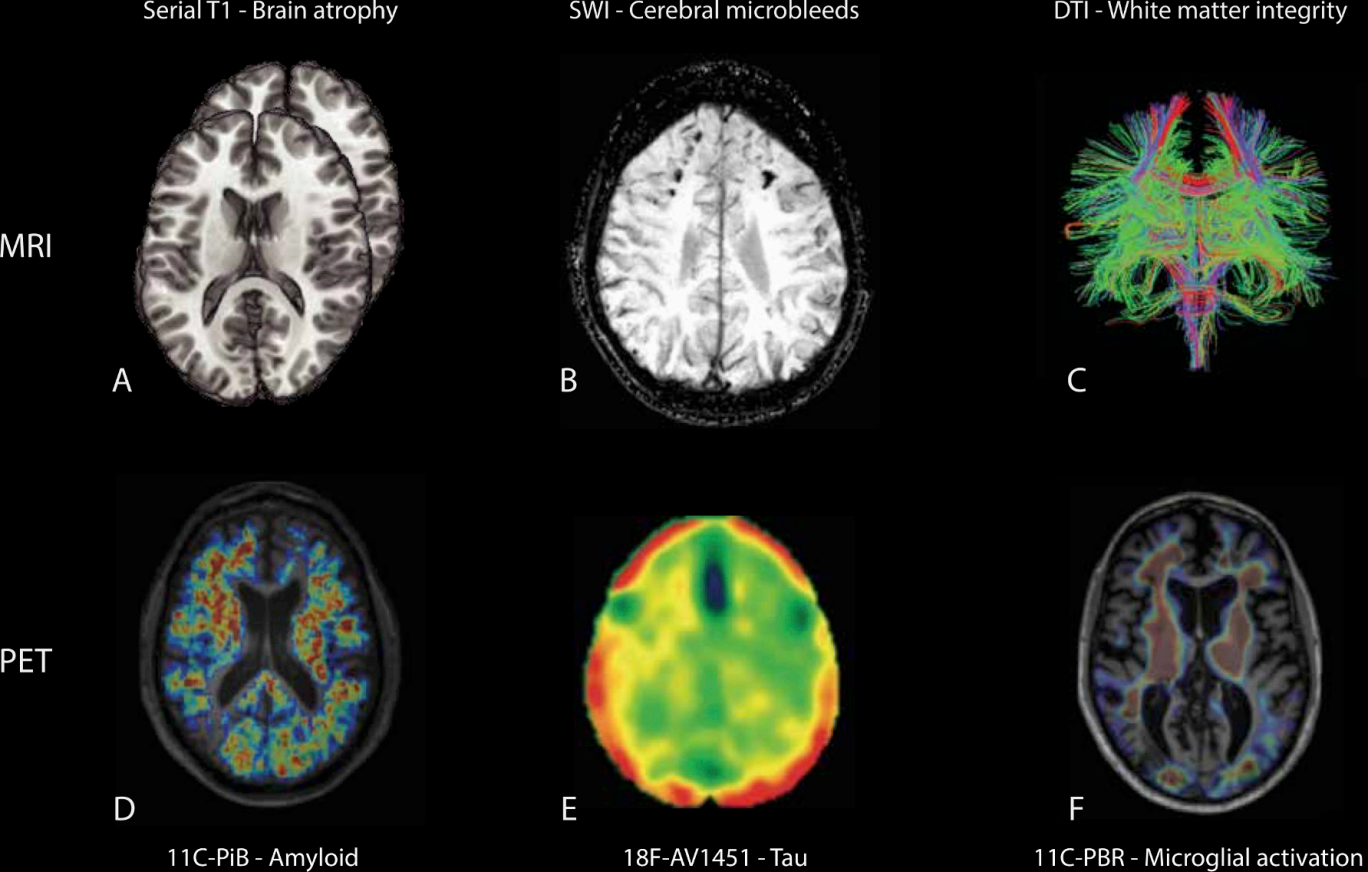
Imaging traumatic brain injury (TBI) and post-traumatic neurodegeneration. (A) Progressive neurodegeneration is quantifiable using repeated T1 MRI used to generate atrophy rates over time. (B) Susceptibility weighted imaging (SWI) shows microbleeds in typical parafalcine distribution, typical of diffuse vascular injuries. (C) Diffusion MRI allows quantification of white matter integrity after axonal injury and provides a measure of diffuse axonal injury.^1^ (D) ^11^C-Pittsburgh compound B (PiB) positron emission tomography (PET) study shows amyloid deposition in a middle-aged woman several years after moderate-severe TBI.^55^ (E) ^18^F-AV1451 tau PET shows abnormal binding following TBI. (F) Persistent abnormal microglial activation on ^11^C-PBR28 translocator protein PET in a middle-aged man a decade after moderate-severe TBI, particularly in white matter regions.^40^ DTI, diffusion tensor imaging.

MRI measures of brain atrophy (figure 5A) and NFL levels provide sensitive but non-specific measures of post-traumatic neurodegeneration. In contrast, molecular imaging techniques such as positron emission tomography (PET) allow specific types of proteinopathy to be identified (figure 5D–F). PET tracers sensitive to hyperphosphorylated tau in neurofibrillary tangles and amyloid beta aggregates have been developed. The application of these in TBI promises to dramatically improve the investigation of post-traumatic neurodegeneration and should facilitate the diagnosis of CTE and other types of post-traumatic dementia in vivo.

Amyloid PET tracers such as ^11^C-Pittsburgh compound-B (^11^C-PiB) have been used widely to identify fibrillar amyloid beta pathology. ^11^C-PiB binding is increased in AD in a similar pattern to amyloid pathology.^s69 s70^ In the first year after TBI, ^11^C-PiB binding is also increased in cortical grey matter and striatum,^s71^ remaining high many years after injury in some patients (figure 5D).^55^ There are similarities between ^11^C-PiB binding in TBI and AD, although binding is typically much higher in AD. Both AD and TBI may lead to increased ^11^C-PiB binding in the posterior cingulate cortex.^s72^ However, after TBI, ^11^C-PiB binding is also seen in the cerebellum, a location where increased amyloid is not typically observed in AD, which may suggest a distinct mechanism for the production of amyloid pathology after TBI. Not all investigators have reported increased amyloid PET signal after TBI, which may be attributable to tracer selection or injury severity.^s6^

Recent work has focused on developing PET tracers specific for hyperphosphorylated tau. The ability to identify tau pathology in vivo is likely to be key to the assessment of CTE, so these developments are of particular interest. A number of tracers appear sensitive and specific to tau pathology in the context of AD. For example, flortaucipir (^18^F-AV1451, ^18^F-T807) shows potent and specific non-displaceable binding to tau neurofibrillary tangles in postmortem AD brain tissue.^s73–75^ Flortaucipir is selective for tau, and does not significantly bind to beta amyloid, alpha-synuclein or TDP-43.^s73^ The pattern of binding relates to clinical phenotype, cognitive profile, and Braak and Braak staging of AD.^s76 s77^ However, in other non-Alzheimer’s tauopathies, the utility of this ligand is less clear.^s75^ Some studies have used tau PET to investigate patients with repetitive TBI produced by sports injuries. A recent study of 26 former American football players with mild cognitive symptoms showed modestly increased flortaucipir binding compared with healthy volunteers.^56^ Tracer binding related to years of participation but not to cognitive performance. The technique was not able to differentiate players from controls at the individual level. Other studies in this area have however been small in size, often lacked controls and have usually lacked neuropathological confirmation of CTE.^s78 s79^ One case report of an NFL player with a history of repetitive TBI and progressive neuropsychiatric symptoms reported increased flortaucipir binding.^s78^ A second case study reported increased ^18^F-FDDNP (2-(1-[6-{(2-[18F]fluoroethyl)(methyl)amino}-2-naphthyl]ethylidene)malononitrile) binding in an NFL player with history of repeated mild TBI who was later diagnosed with CTE postmortem. In this case, the spatial pattern of abnormal PET findings correlated to some extent with the spatial pattern of P-tau postmortem.^s80^ Current studies exploring flortaucipir and other PET ligands should clarify whether tau PET will be diagnostically useful following TBI (figure 5E).

PET scanning also provides a way to investigate other processes associated with neurodegeneration. For example, activated microglia often colocate with amyloid and tau pathology. ^11^C-PBR28 translocator protein (TSPO) PET ligands bind to a translocator protein expressed on the mitochondria of activated microglia.^s81^ These have been widely used in AD, generally showing increased binding that tracks progression of the disease.^s82^ In neuropathological studies of TBI, chronic microglial activation is associated with evidence of persistent axonal injury (figure 5F).^28^ In keeping with these observations, TSPO PET binding is increased many years after TBI, predominantly in subcortical white matter and thalamic locations.^39 40^ High binding is seen in areas of diffuse axonal injury that also show progressive brain atrophy.^40^ Hence, microglial activation persists in areas of axonal injury for years after TBI and progressive neurodegeneration occurs at these locations. A key issue is what functional role these chronically activated microglia play. TSPO PET does not distinguish between distinct microglial phenotypes, so activated microglia in this context might be inflammatory or restorative.^s83^ Using an experimental medicine approach, we combined pharmacological intervention, neuroimaging and fluid biomarkers to investigate this issue. Minocycline was used to inhibit chronically activated microglia, an effect confirmed by reductions in TSPO binding after treatment. Serially monitored NFL blood levels provided a dynamic measure of neurodegenerative activity. Increases in NFL were seen following minocycline treatment,^40^ providing evidence inhibiting microglia increases neurodegeneration in the chronic phase after TBI. This suggests that microglia may have a restorative function late after TBI, in keeping with work in non-human primates showing a trophic role for chronically activated microglia.^s37^

### Vascular damage

Neurodegenerative abnormalities are particularly seen in a perivascular location, suggesting that the TBI may trigger the neurodegenerative cascade through an effect on BBB permeability.^22 31^ Hence, investigating neurovascular structures could provide insights into the triggers for neurodegeneration. Blood vessels are often directly damaged by TBI. Large intracerebral haemorrhages are common in extradural, subdural and parenchymal locations. These are often the focus for initial management. However, the relationship of large haemorrhage to long-term dementia risk is unclear. In other contexts, such as intraparenchymal or non-traumatic subarachnoid haemorrhage, long-term dementia risk is elevated independent of vascular risk factors: dementia risk is significantly greater following haemorrhagic compared with ischaemic stroke.^s84^ Following TBI, more subtle perivascular haemorrhage is also common and can be sensitively assessed using gradient echo or susceptibility weighted imaging.^s85^ These MRI sequences are now routinely used in the assessment of TBI, and microhaemorrhages provide evidence of diffuse vascular injury, which may not be apparent using conventional imaging approaches (figure 5B). Diffuse vascular injury is often associated, but is not synonymous with, diffuse axonal injury, and the location and extent of microhaemorrhages may be another way to investigate the link between initial injury severity and post-traumatic neurodegeneration.

TBI can also produce non-haemorrhagic disruption of the neurovascular unit. BBB permeability is increased acutely after TBI, but the duration of this change is uncertain.^s86^ In humans, neuroimaging developments allow subtle disruption of BBB permeability to be identified. For example, dynamic contrast-enhanced (DCE) MRI with fast T1 mapping has been used to identify subtle changes in BBB permeability in healthy ageing, early neurodegenerative conditions^s87^ and American football players during a season of play.^s88^ This approach shows considerable promise. DCE changes correlate with BBB damage on molecular imaging and can distinguish patients with TBI from healthy controls.^s89 s90^ Recent preclinical work in mild closed TBI validates DCE as a measure of non-haemorrhagic BBB disruption.^31^ Future work would usefully study BBB permeability in the chronic phase postinjury and clarify its relationship to brain atrophy and associated proteinopathies.

## Post-traumatic neurodegeneration and clinical trials

Establishing clear relationships between biomarkers and disease states can facilitate the development of new treatments. For example, treatment advances have been accelerated by establishing the links between intraocular pressure and visual function in glaucoma; bone mineral density and osteoporotic fractures; and CD4 lymphocyte count for clinical outcome in HIV infection. This consideration is relevant for TBI because there is a pressing need to develop new approaches to the evaluation of new treatments.^s91^ The heterogeneity of TBI leads to significant challenges in powering clinical trials.^s91^ Previous studies of neuroprotection and disease modification have largely produced negative results. However, these trials were often underpowered to detect treatment effects that were usually measured using noisy clinical endpoints such as the Glasgow Outcome Scale. Hence, there is a significant risk that we have failed to properly evaluate promising new treatments because of suboptimal trial design.

**Figure 6:**
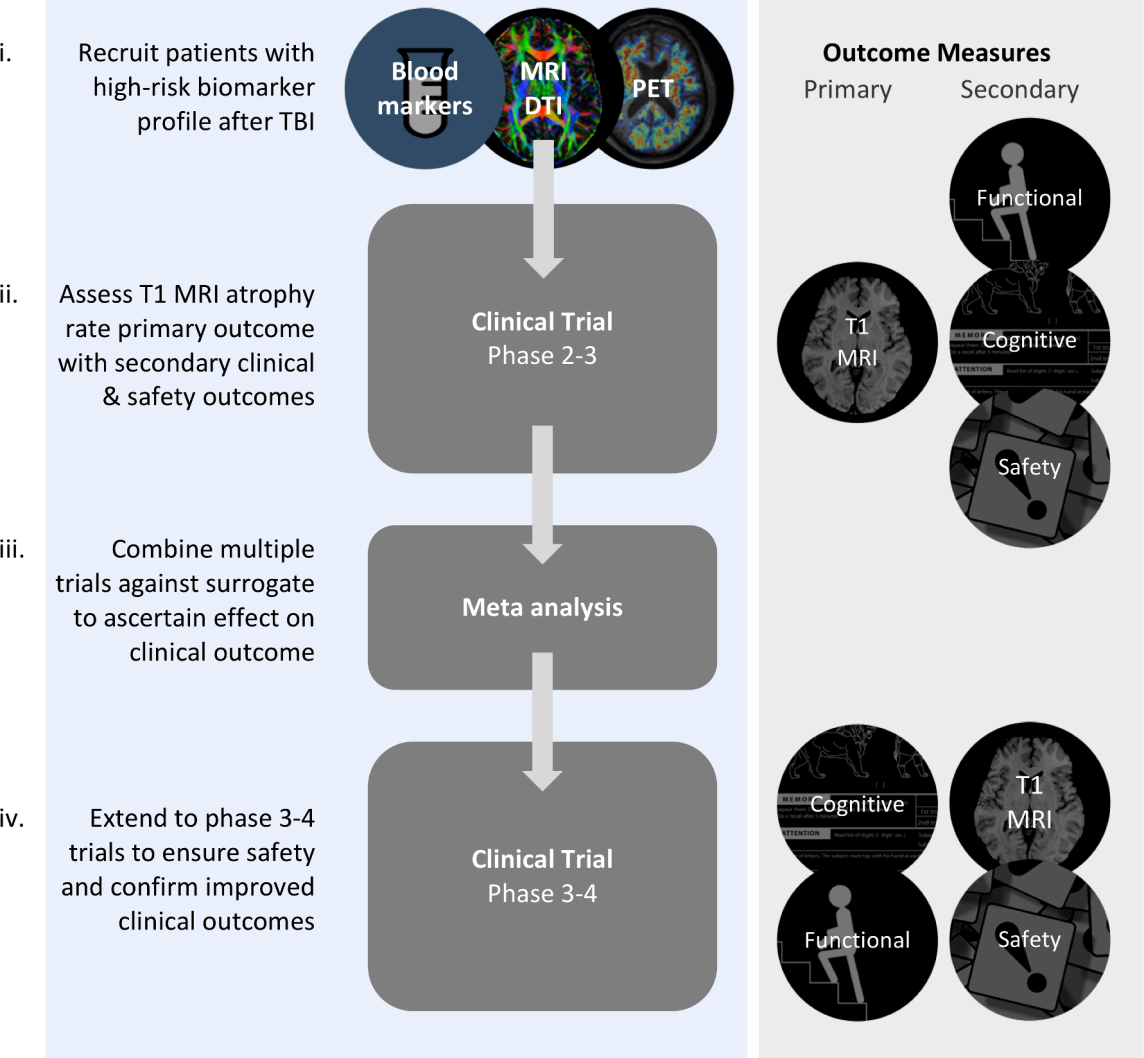
Biomarkers in clinical trials after traumatic brain injury (TBI). Stages for the evaluation of disease-modifying/neuroprotective treatment after TBI. (i) Recruitment of patients at high risk for neurodegeneration using baseline blood neurofilament light, diffusion tensor imaging abnormality (DTI) and positron emission tomography (PET) abnormality. (ii) Phase 2–3 trials powered to primary outcome measure of change in atrophy rate (using repeated T1 MRI) with secondary functional/cognitive/safety outcomes. (iii) Meta-analysis of phase 2–3 trials to clarify the relationship between the surrogate (T1 atrophy rate) and patient-centred outcomes. (iv) Late-stage phase 3–4 trials using primary functional or cognitive outcome. This may be a composite measure.

Incorporating biomarkers of neurodegeneration as primary or secondary outcome measures in phase II and III clinical trials will improve the ability to detect neuroprotective and disease-modifying treatments by providing precise and sensitive measures of neuronal loss (figure 6). Fluid biomarkers such as NFL provide a potential surrogate marker of neurodegeneration. For example, changes in blood or CSF levels of NFL can be used as a dynamic measure of treatment effects that have been validated in animal studies^51^ and show promising results as a read-out of active neurodegeneration in our experimental medicine study of minocycline use.^40^ MRI measurements of brain atrophy provide a second option with strong face validity as an integrated measure of neuronal loss and an established link with neuropathology in other contexts such as AD.^57^ Brain atrophy is established for a variety of neurodegenerative conditions, with clinical trials increasingly incorporating it as a surrogate endpoint for disease progression.^s91^

MRI measurements of atrophy have high test–retest reliability in healthy subjects, allowing the impact of TBI to be sensitively detected. The effect of TBI on brain atrophy is substantial in comparison with a number of potential confounds. For example, TBI explained ~20% of the variance in atrophy rates compared with ~0.5% due to either age or sex in our recent study.^43^ Hence, relatively small treatment effects on atrophy rate could be identified using this approach.^43^ ^s92 s93^ Using the most sensitive imaging measure of atrophy (Jacobian determinant rate), we established that groups of ~200 patients per trial arm were necessary to detect reductions of atrophy that are likely to be meaningful (box 2).^43^ Sample size could be reduced by enriching for high rates of neurodegeneration using complementary biomarkers, an approach routinely taken in AD trials.^58^ In TBI, this type of enrichment might involve inclusion criteria such as positive tau PET, the presence of diffuse axonal injury indicated by DTI and high levels of plasma/CSF NFL (figure 3). This approach would facilitate cost-effective and feasible phase II clinical trials, providing robust evidence about the effect of neuroprotective or disease-modifying treatments on neuronal loss after TBI.

Box 2 **Sample size in clinical trials of post-traumatic dementia therapeutics**

- The evaluation of new treatments in traumatic brain injury (TBI) could be accelerated by adopting biomarkers of neurodegeneration as outcome measures. The Food and Drug Administration approval of biomarkers of disease progression in Alzheimer’s disease (AD) will allow new treatments that target post-traumatic neurodegeneration to be used in clinical trials. Brain atrophy measured by MRI is the most promising biomarker in this respect.
- There are important advantages in using imaging biomarkers from a trial design perspective. The ‘effect size’ (ES) of any treatment is key contributor to sample size (N), and the variability of a study’s main outcome measure is a major determinant of the ES (see equations below). Using a biomarker such as brain atrophy rate with high test–retest reliability reduces the variance and increases ES, driving down the required sample size. Likewise, increasing the treatment effectiveness (the difference between the average atrophy rates in the treatment and control groups) also increases ES and reduces sample size. The ES could be further increased by recruiting patients likely to respond to treatment (‘enriching the population’). Those at high neurodegenerative risk might be identified using biomarkers such as blood neurofilament light, genetic risk, diffusion tensor imaging, positron emission tomography imaging or baseline atrophy.^43^
- The sample size calculation also reflects investigator choices about the acceptable risk of errors: the lower the acceptable error rate, the higher the N required. These choices manifest in critical values (z, derived from a standard normal distribution) seen in the numerator of the sample size equation. The z values must be inputted for the risk of type I errors (α), usually set at 5% and for type II errors (1–β), where the power (β) is often set at 80%.^s104^ The size of the treatment effect felt to be clinically meaningful is included in the denominator: in the AD literature a reduction of 25% in atrophy rate is felt to be clinically significant. This is a pragmatic starting point in TBI trials.^s105^ Using white matter atrophy rate as a primary outcome measure, an α of 5% and β of 80%, 200 participants per arm are needed to identify a 25% reduction atrophy in the chronic phase of TBI.^43^ This does not include any enrichment of the study population, which might bring down the sample size further.

­

$effectsize=\frac{meanatrophyrate_{patients}-meanatrophyrate_{controls}}{\sqrt{varianceinatrophyrate_{patients}}}$

­

$samplesize=2\times {\left[\frac{criticalvalue{\left(z\right)}_{\frac{\alpha }{2}}+criticalvalue{\left(z\right)}_{1-\beta }}{treatmenteffect\times effectsize}\right]}^{2}$

Adapted from Cash *et al*.^59^

There are a number of complexities that need to be considered when using MRI measures of brain atrophy following TBI. Injury-related oedema produced in the acute phase may spuriously elevate atrophy rates as it resolves. A similar issue is recognised in multiple sclerosis following the initiation of treatments which reduce neuroinflammation (‘pseudo-atrophy’).^s94^ Studies that accurately characterise the time course of atrophy using repeated MRI early after TBI are necessary to clarify how best to precisely measure neuronal loss and distinguish it from resolving oedema and any treatment-related effects.^s95 s96^ A methodologically simpler approach is to measure atrophy in the subacute-to-chronic phase, following the resolution of acute oedema. Brain atrophy progresses months to years after injuries and so atrophy can provide an integrative measure of neuronal loss over time. This may prove to be a sensitive measure of treatment effects that were administered in the acute phase prior to MRI assessment. Regardless of the timing of assessment, focal lesions need to be controlled for in the estimation of atrophy rates. This can be achieved by delineating focal lesions and then excluding areas of obvious damage from the calculation of atrophy rates. This also allows investigation of the spatial relationship between atrophy and focal injury.^s97 s98^ Other non-TBI-specific factors that need to be considered include hydration status, motion, scanner variability and harmonisation of analysis technique.^59^ ^s99^^–^^101^

Another important issue is to understand how atrophy rates relate to clinical outcomes after TBI. While clinical outcomes such as quality of life and disability are ultimately key to evaluating new treatments, there is a complex relationship between brain atrophy and these clinical measures. This is generally true for neurodegenerative conditions, but the relationship is likely to be particularly complex following TBI. Patients with high rates of atrophy have been shown to have worse functional outcomes and cognitive impairment,^43 44^ ^s57^ but more work is necessary to properly characterise these relationships. One issue is that spontaneous recovery early after TBI drives much of the early clinical change, so neurodegeneration triggered by the injury is unlikely to show a clear relationship to these outcomes. For example, cognitive function generally improves over the first few months after TBI as a result of spontaneous recovery, supported by early neurorehabilitation intervention.^s102^ High rates of brain atrophy are seen during this period, but any treatment effect on cognition is likely to be obscured by this spontaneous recovery occurring (figure 1A). Hence, directly studying the effects of neuroprotective interventions on cognitive function early after TBI is likely to be confounded by the competing effects of distinct neurodegenerative and recovery processes. A second issue is that the effects of accelerated neurodegeneration may take years if not decades to become apparent. A young person with accelerated neurodegeneration after TBI is likely to have a large neural and cognitive reserve to protect against the impact of neuronal loss. Hence the cumulative effects of progressive neuronal loss may not become apparent clinically until many years have elapsed (figure 1A), by which time the link to a previous injury may not be appreciated.

Similar issues are important for the design of early-stage dementia studies, where preclinical drug effects may be disease-modifying but would not be expected to have immediate clinical effects (figure 6).^s103^ In early 2018 the US Food and Drug Administration (FDA) released draft guidance for the assessment of early AD treatments.^60^ This recognised the challenges of assessing treatments in high-risk individuals without cognitive or functional impairments, a preclinical period they term ‘stage 1’ of the disease. Significantly, the FDA accepted that biomarker changes may be sufficient grounds for initial approval, with postmarketing surveillance:

‘In Stage 1 patients, an effect on the characteristic pathophysiologic changes of AD, as demonstrated by an effect on various biomarkers, may be measured. Such an effect, analysed as a primary efficacy measure, may, in principle, serve as the basis for an accelerated approval (ie, the biomarker effects would be found to be reasonably likely to predict clinical benefit, with a post-approval requirement for a study to confirm the predicted clinical benefit). As with the use of neuropsychological tests, a pattern of treatment effects seen across multiple individual biomarker measures would increase the persuasiveness of the putative effect’.^60^

These considerations have important implications for the design of clinical trials in TBI. One option, considering the FDA’s draft guidance for AD, might be to apply brain atrophy and fluid biomarker measures of neuronal loss as a primary endpoint for the initial evaluation of neuroprotective treatment, later making use of compound functional and cognitive outcome scores for postmarketing studies. This approach would recognise that changes in neurodegenerative biomarkers such as brain atrophy provide a legitimate initial treatment goal, while incorporating clinical assessments once treatment effectiveness has been established.

## Conclusions

There is a well-established link between TBI and dementia, which is increasingly understood at the mechanistic level. Post-traumatic neurodegeneration is common, but it is unclear how to diagnose distinct types of post-traumatic dementia in clinical practice. This is important because significant TBI contributes to the population burden of dementia. Around 5% of all dementia cases may be attributable to TBI, assuming a conservative 1.5 times increase in relative risk for dementia postinjury.^8^ Conversely, many individuals are anxious about the long-term impact of very minor head injuries, which are unlikely to have long-term effects and should be differentiated from the more concerning situation following repeated mild or single moderate-severe TBI. The systematic evaluation and prospective validation of neuroimaging and fluid biomarker measures of neurodegeneration promises to define the endophenotypes of post-traumatic dementias. This has the potential to dramatically improve the diagnosis of post-traumatic dementias and allow more efficient clinical trials in TBI by enriching trial populations for high levels of progressive neurodegeneration and by providing sensitive outcome measures of neuronal loss.

Additional references can be found in the online supplementary file.

10.1136/jnnp-2017-317557.supp1 Supplementary data
