# Supplementary material for: Understanding neurodegeneration after traumatic brain injury: from mechanisms to clinical trials in dementia
Source: J Neurol Neurosurg Psychiatry. 2019 Sep 21;90(11):1221–33. doi: 10.1136/jnnp-2017-317557 (PMC6860906; doi:10.1136/jnnp-2017-317557)
Supplement: Supplementary data [file jnnp-2017-317557supp001.pdf]

Understanding neurodegeneration after traumatic brain injury: from mechanisms to clinical trials in dementia

SUPPLEMENTARY REFERENCES

s1. Evans SA, Airey MC, Chell SM, et al. Disability in young adults following major trauma: 5 year follow up of survivors. BMC Public Health 2003;3:8.

s2. Xu W, Tan L, Wang HF, et al. Meta-analysis of modifiable risk factors for Alzheimer's disease. J Neurol Neurosurg Psychiatry 2015;86(12):1299-306

s3. Godbolt AK, Cancelliere C, Hincapie CA, et al. Systematic review of the risk of dementia and chronic cognitive impairment after mild traumatic brain injury: results of the International Collaboration on Mild Traumatic Brain Injury Prognosis. Arch Phys Med Rehabil 2014;95(3 Suppl):S245-56. doi: 10.1016/j.apmr.2013.06.036

s4. Bazarian JJ, Cernak I Fau - Noble-Haeusslein L, Noble-Haeusslein L Fau - Potolicchio S, et al. Long-term neurologic outcomes after traumatic brain injury. J Head Trauma Rehabil 2009;24(1550-509X (Electronic)):439-51.

s5. Tripodis Y, Alosco ML, Zirogiannis N, et al. The Effect of Traumatic Brain Injury History with Loss of Consciousness on Rate of Cognitive Decline Among Older Adults with Normal Cognition and Alzheimer's Disease Dementia. J Alzheimers Dis 2017;59(1):251-63. doi: 10.3233/JAD-160585

s6. Weiner MW, Crane PK, Montine TJ, et al. Traumatic brain injury may not increase the risk of Alzheimer disease. Neurology 2017;89(18):1923-25. doi: 10.1212/WNL.0000000000004608

s7. Sugarman MA, McKee AC, Stein TD, et al. Failure to detect an association between self-reported traumatic brain injury and Alzheimer's disease neuropathology and dementia. Alzheimer’s Dement 2019 doi: 10.1016/j.jalz.2018.12.015

s8. Chen H, Richard M, Sandler DP, et al. Head injury and amyotrophic lateral sclerosis. Am J Epidemiol 2007;166(7):810-6.

s9. Armon C, Nelson LM. Is head trauma a risk factor for amyotrophic lateral sclerosis? An evidence based review. Amyotroph Lateral Scler 2012;13(4):351-6.

s10. Chio A, Benzi G, Dossena M, et al. Severely increased risk of amyotrophic lateral sclerosis among Italian professional football players. Brain 2005;128(Pt 3):472-6.

s11. Chio A, Calvo A, Dossena M, et al. ALS in Italian professional soccer players: the risk is still present and could be soccer-specific. Amyotroph Lateral Scler 2009;10(4):205-9.

s12. Armon C. Sports and trauma in amyotrophic lateral sclerosis revisited. J Neurol Sci 2007;262(1-2):45-53.

s13. Lehman EJ, Hein MJ, Baron SL, et al. Neurodegenerative causes of death among retired National Football League players. Neurology 2012;79(19):1970-4.

s14. Savica R, Parisi JE, Wold LE, et al. High school football and risk of neurodegeneration: a community-based study. Mayo Clin Proc 2012;87(4):335-40.

s15. Deutsch MB, Mendez MF, Teng E. Interactions between traumatic brain injury and frontotemporal degeneration. Dement Geriatr Cogn Disord 2015;39(3-4):143-53.

s16. Wang HK, Lee YC, Huang CY, et al. Traumatic brain injury causes frontotemporal dementia and TDP-43 proteolysis. Neuroscience 2015;300:94-103.

s17. Rosso SM, Landweer EJ, Houterman M, et al. Medical and environmental risk factors for sporadic frontotemporal dementia: a retrospective case-control study. J Neurol Neurosurg Psychiatry 2003;74(11):1574-6.

s18. Kalkonde YV, Jawaid A, Qureshi SU, et al. Medical and environmental risk factors associated with frontotemporal dementia: a case-control study in a veteran population. Alzheimers Dement 2012;8(3):204-10.

s19. LoBue C, Wilmoth K, Cullum CM, et al. Traumatic brain injury history is associated with earlier age of onset of frontotemporal dementia. J Neurol Neurosurg Psychiatry 2016;87(8):817-20.

s20. Bieniek KF, Ross OA, Cormier KA, et al. Chronic traumatic encephalopathy pathology in a neurodegenerative disorders brain bank. Acta Neuropathol 2015;130(6):877-89.

s21. Perry DC, Sturm VE, Peterson MJ, et al. Association of traumatic brain injury with subsequent neurological and psychiatric disease: a meta-analysis. J Neurosurg 2016;124(2):511-26.

s22. Zeng S, Jiang JX, Xu MH, et al. Prognostic value of apolipoprotein E epsilon4 allele in patients with traumatic brain injury: a meta-analysis and meta-regression. Genet Test Mol Biomarkers 2014;18(3):202-10.

s23. Hayes JP, Logue MW, Sadeh N, et al. Mild traumatic brain injury is associated with reduced cortical thickness in those at risk for Alzheimer's disease. Brain 2017;140(3):813-25.

s24. Albayram O, Kondo A, Mannix R, et al. Cis P-tau is induced in clinical and preclinical brain injury and contributes to post-injury sequelae. Nat Commun 2017;8(1):1000. doi: 10.1038/s41467-017-01068-4

s25. Johnson VE, Stewart W, Trojanowski JQ, et al. Acute and chronically increased immunoreactivity to phosphorylation-independent but not pathological TDP-43 after a single traumatic brain injury in humans. Acta Neuropathol 2011;122(6):715-26. doi: 10.1007/s00401-011-0909-9

s26. Roberts GW, Gentleman SM, Lynch A, et al. beta A4 amyloid protein deposition in brain after head trauma. Lancet 1991;338(8780):1422-3.

s27. Schmidt ML, Zhukareva V, Newell KL, et al. Tau isoform profile and phosphorylation state in dementia pugilistica recapitulate Alzheimer's disease. Acta Neuropathol 2001;101(5):518-24.

s28. Maxwell WL, Bartlett E, Morgan H. Wallerian degeneration in the optic nerve stretch-injury model of traumatic brain injury: a stereological analysis. J Neurotrauma 2015;32(11):780-90.

s29. Hill CS, Coleman MP, Menon DK. Traumatic Axonal Injury: Mechanisms and Translational Opportunities. Trends in neurosciences 2016;39(5):311-24.

s30. Uryu K, Chen XH, Martinez D, et al. Multiple proteins implicated in neurodegenerative diseases accumulate in axons after brain trauma in humans. Exp Neurol 2007;208(2):185-92.

s31. Jucker M, Walker LC. Self-propagation of pathogenic protein aggregates in neurodegenerative diseases. Nature 2013;501(7465):45-51.

s32. Nakamura K, Greenwood A, Binder L, et al. Proline isomer-specific antibodies reveal the early pathogenic tau conformation in Alzheimer's disease. Cell 2012;149(1):232-44.

s33. Yang Z, Lin F, Robertson CS, et al. Dual vulnerability of TDP-43 to calpain and caspase-3 proteolysis after neurotoxic conditions and traumatic brain injury. J Cereb Blood Flow Metab 2014;34(9):1444-52.

s34. McKee AC, Gavett BE, Stern RA et al (2010) TDP-43 proteinopathy and motor neuron disease in chronic traumatic encephalopathy. J Neuropathol Exp Neurol 69:918–929

s35. Csuka E, Morganti-Kossmann MC, Lenzlinger PM, et al. IL-10 levels in cerebrospinal fluid and serum of patients with severe traumatic brain injury: relationship to IL-6, TNF-alpha, TGF-beta1 and blood-brain barrier function. Journal of neuroimmunology 1999;101(2):211-21.

s36. Shapira Y, Setton D, Artru AA, et al. Blood-brain barrier permeability, cerebral edema, and neurologic function after closed head injury in rats. Anesthesia and analgesia 1993;77(1):141-8.

s37. Nagamoto-Combs K, McNeal DW, Morecraft RJ, et al. Prolonged microgliosis in the rhesus monkey central nervous system after traumatic brain injury. J Neurotrauma 2007;24(11):1719-42.

s38. Koshinaga M, Katayama Y, Fukushima M, et al. Rapid and widespread microglial activation induced by traumatic brain injury in rat brain slices. J Neurotrauma 2000;17(3):185-92.

s39. Ransohoff RM. A polarizing question: do M1 and M2 microglia exist? Nat Neurosci 2016;19(8):987-91.

s40. Iliff JJ, Wang M, Liao Y, et al. A Paravascular Pathway Facilitates CSF Flow Through the Brain Parenchyma and the Clearance of Interstitial Solutes, Including Amyloid β. Science Translational Medicine 2012;4(147):147ra11.

s41. Clavaguera F, Hench J, Goedert M, et al. Invited review: Prion-like transmission and spreading of tau pathology. Neuropathol Appl Neurobiol 2015;41(1):47-58.

s42. Kenney K, Iacono D, Edlow BL, et al. Dementia After Moderate-Severe Traumatic Brain Injury: Coexistence of Multiple Proteinopathies. J Neuropathol Exp Neurol 2018;77(1):50-63. doi: 10.1093/jnen/nlx101

s43. Crary JF, Trojanowski JQ, Schneider JA, et al. Primary age-related tauopathy (PART): a common pathology associated with human aging. Acta Neuropathol 2014;128(6):755-66. doi: 10.1007/s00401-014-1349-0

s44. Kovacs GG, Ferrer I, Grinberg LT, et al. Aging-related tau astrogliopathy (ARTAG): harmonized evaluation strategy. Acta Neuropathol 2016;131(1):87-102. doi: 10.1007/s00401-015-1509-x

s45. Stewart W, Allinson K, Al-Sarraj S, et al. Primum non nocere: a call for balance when reporting on CTE. Lancet Neurol 2019;18(3):231-33. doi: 10.1016/s1474-4422(19)30020-1

s46. Iacono WG. Endophenotypes in psychiatric disease: prospects and challenges. Genome medicine 2018;10(1):11-11.

s47. Edlow BL, Keene CD, Perl DP, et al. Multimodal Characterization of the Late Effects of Traumatic Brain Injury: A Methodological Overview of the Late Effects of Traumatic Brain Injury Project. J Neurotrauma 2018;35(14):1604-19. doi: 10.1089/neu.2017.5457

s48. Worker A, Dima D, Combes A, et al. Test-retest reliability and longitudinal analysis of automated hippocampal subregion volumes in healthy ageing and Alzheimer's disease populations. Hum Brain Mapp 2018;39(4):1743-54.

s49. Reuter M, Schmansky NJ, Rosas HD, et al. Within-subject template estimation for unbiased longitudinal image analysis. Neuroimage 2012;61(4):1402-18. doi: 10.1016/j.neuroimage.2012.02.084

s50. Smith SM, Rao A, De Stefano N, et al. Longitudinal and cross-sectional analysis of atrophy in Alzheimer's disease: cross-validation of BSI, SIENA and SIENAX. Neuroimage 2007;36(4):1200-6.

s51. Brezova V, Moen KG, Skandsen T, et al. Prospective longitudinal MRI study of brain volumes and diffusion changes during the first year after moderate to severe traumatic brain injury. Neuroimage Clin 2014;5:128-40.

s52. Bendlin BB, Ries ML, Lazar M, et al. Longitudinal changes in patients with traumatic brain injury assessed with diffusion-tensor and volumetric imaging. Neuroimage 2008;42(2):503-14.

s53. MacKenzie JD, Siddiqi F, Babb JS, et al. Brain atrophy in mild or moderate traumatic brain injury: a longitudinal quantitative analysis. AJNR Am J Neuroradiol 2002;23(9):1509-15.

s54. Trivedi MA, Ward MA, Hess TM, et al. Longitudinal changes in global brain volume between 79 and 409 days after traumatic brain injury: relationship with duration of coma. J Neurotrauma 2007;24(5):766-71.

s55. Zhou Y, Kierans A, Kenul D, et al. Mild traumatic brain injury: longitudinal regional brain volume changes. Radiology 2013;267(3):880-90.

s56. Warner MA, Marquez de la Plata C, Spence J, et al. Assessing spatial relationships between axonal integrity, regional brain volumes, and neuropsychological outcomes after traumatic axonal injury. J Neurotrauma 2010;27(12):2121-30.

s57. Ross DE, Ochs AL, Seabaugh JM, et al. Progressive brain atrophy in patients with chronic neuropsychiatric symptoms after mild traumatic brain injury: a preliminary study. Brain Inj 2012;26(12):1500-9.

s58. Jones DK, Cercignani M. Twenty-five pitfalls in the analysis of diffusion MRI data. NMR Biomed 2010;23(7):803-20. doi: 10.1002/nbm.1543

s59. Warren Jason D, Rohrer Jonathan D, Hardy J. Disintegrating Brain Networks: from Syndromes to Molecular Nexopathies. Neuron 2012;73(6):1060-62.

s60. Kuhle J, Barro C, Andreasson U, et al. Comparison of three analytical platforms for quantification of the neurofilament light chain in blood samples: ELISA, electrochemiluminescence immunoassay and Simoa. Clin Chem Lab Med 2016;54(10):1655-61.

s61. Gaiottino J, Norgren N, Dobson R, et al. Increased neurofilament light chain blood levels in neurodegenerative neurological diseases. PLoS One 2013;8(9):e75091.

s62. Lu CH, Macdonald-Wallis C, Gray E, et al. Neurofilament light chain: A prognostic biomarker in amyotrophic lateral sclerosis. Neurology 2015;84(22):2247-57.

s63. Gisslen M, Price RW, Andreasson U, et al. Plasma Concentration of the Neurofilament Light Protein (NFL) is a Biomarker of CNS Injury in HIV Infection: A Cross-Sectional Study. EBioMedicine 2016;3:135-40.

s64. Hansson O, Janelidze S, Hall S, et al. Blood-based NfL: A biomarker for differential diagnosis of parkinsonian disorder. Neurology 2017;88(10):930-37.

s65. Shahim P, Zetterberg H, Tegner Y, et al. Serum neurofilament light as a biomarker for mild traumatic brain injury in contact sports. Neurology 2017;88(19):1788-94.

s66. Neselius S, Zetterberg H, Blennow K, et al. Olympic boxing is associated with elevated levels of the neuronal protein tau in plasma. Brain Inj 2013;27(4):425-33.

s67. Shahim P, Tegner Y, Marklund N, et al. Neurofilament light and tau as blood biomarkers for sports-related concussion. Neurology 2018;90(20):e1780-e88.

s68. Ljungqvist J, Zetterberg H, Mitsis M, et al. Serum Neurofilament Light Protein as a Marker for Diffuse Axonal Injury: Results from a Case Series Study. J Neurotrauma 2017;34(5):1124-27.

s69. Quigley H, Colloby SJ, O'Brien JT. PET imaging of brain amyloid in dementia: a review. Int J Geriatr Psychiatry 2011;26(10):991-9.

s70. Rowe CC, Ng S, Ackermann U, et al. Imaging beta-amyloid burden in aging and dementia. Neurology 2007;68(20):1718-25.

s71. Hong YT, Veenith T, Dewar D, et al. Amyloid imaging with carbon 11-labeled Pittsburgh compound B for traumatic brain injury. JAMA Neurol 2014;71(1):23-31.

s72. Tosun D, Schuff N, Mathis CA, et al. Spatial patterns of brain amyloid-beta burden and atrophy rate associations in mild cognitive impairment. Brain 2011;134(Pt 4):1077-88.

s73. Marquie M, Normandin MD, Vanderburg CR, et al. Validating novel tau positron emission tomography tracer [F-18]-AV-1451 (T807) on postmortem brain tissue. Ann Neurol 2015;78(5):787-800.

s74. Sander K, Lashley T, Gami P, et al. Characterization of tau positron emission tomography tracer [(18)F]AV-1451 binding to postmortem tissue in Alzheimer's disease, primary tauopathies, and other dementias. Alzheimers Dement 2016;12(11):1116-24.

s75. Marquie M, Normandin MD, Meltzer AC, et al. Pathological correlations of [F-18]-AV-1451 imaging in non-alzheimer tauopathies. Ann Neurol 2017;81(1):117-28.

s76. Ossenkoppele R, Schonhaut DR, Scholl M, et al. Tau PET patterns mirror clinical and neuroanatomical variability in Alzheimer's disease. Brain 2016;139(Pt 5):1551-67.

s77. Schwarz AJ, Yu P, Miller BB, et al. Regional profiles of the candidate tau PET ligand 18F-AV-1451 recapitulate key features of Braak histopathological stages. Brain 2016;139(Pt 5):1539-50.

s78. Dickstein DL, Pullman MY, Fernandez C, et al. Cerebral [(18) F]T807/AV1451 retention pattern in clinically probable CTE resembles pathognomonic distribution of CTE tauopathy. Transl Psychiatry 2016;6(9):e900.

s79. Barrio JR, Small GW, Wong KP, et al. In vivo characterization of chronic traumatic encephalopathy using [F-18]FDDNP PET brain imaging. Proc Natl Acad Sci U S A 2015;112(16):E2039-47.

s80. Omalu B, Small GW, Bailes J, et al. Postmortem Autopsy-Confirmation of Antemortem [F-18]FDDNP-PET Scans in a Football Player With Chronic Traumatic Encephalopathy. Neurosurgery 2018;82(2):237-46.

s81. Owen DR, Guo Q, Kalk NJ, et al. Determination of [11C]PBR28 Binding Potential in vivo: A First Human TSPO Blocking Study. Journal of Cerebral Blood Flow & Metabolism 2014;34(6):989-94.

s82. Lagarde J, Sarazin M, Bottlaender M. In vivo PET imaging of neuroinflammation in Alzheimer’s disease. Journal of Neural Transmission 2018;125(5):847-67.

s83. Perry VH, Nicoll JAR, Holmes C. Microglia in neurodegenerative disease. Nature Reviews Neurology 2010;6:193.

s84. Corraini P, Henderson VW, Ording AG, et al. Long-Term Risk of Dementia Among Survivors of Ischemic or Hemorrhagic Stroke. Stroke 2017;48(1):180-86.

s85. Scheid R, Preul C, Gruber O, et al. Diffuse axonal injury associated with chronic traumatic brain injury: evidence from T2*-weighted gradient-echo imaging at 3 T. AJNR Am J Neuroradiol 2003;24(6):1049-56.

s86. Hay JR, Johnson VE, Young AM, et al. Blood-Brain Barrier Disruption Is an Early Event That May Persist for Many Years After Traumatic Brain Injury in Humans. J Neuropathol Exp Neurol 2015;74(12):1147-57.

s87. Taheri S, Gasparovic C, Shah NJ, et al. Quantitative measurement of blood-brain barrier permeability in human using dynamic contrast-enhanced MRI with fast T1 mapping. Magn Reson Med 2011;65(4):1036-42.

s88. Weissberg I, Veksler R, Kamintsky L, et al. Imaging blood-brain barrier dysfunction in football players. JAMA Neurol 2014;71(11):1453-5.

s89. Winter C, Bell C, Whyte T, et al. Blood-brain barrier dysfunction following traumatic brain injury: correlation of K(trans) (DCE-MRI) and SUVR (99mTc-DTPA SPECT) but not serum S100B. Neurol Res 2015;37(7):599-606.

s90. Yoo RE, Choi SH, Oh BM, et al. Quantitative dynamic contrast-enhanced MR imaging shows widespread blood-brain barrier disruption in mild traumatic brain injury patients with post-concussion syndrome. Eur Radiol 2018

s91. Narayan RK, Michel ME, Ansell B, et al. Clinical trials in head injury. J Neurotrauma 2002;19(5):503-57.

s92. Cash DM, Rohrer JD, Ryan NS, et al. Imaging endpoints for clinical trials in Alzheimer's disease. Alzheimers Res Ther 2014;6(9):87.

s93. Maclaren J, Han Z, Vos SB, et al. Reliability of brain volume measurements: a test-retest dataset. Sci Data 2014;1:140037.

s94. Arnold DL, De Stefano N. Preventing brain atrophy should be the gold standard of effective therapy in multiple sclerosis (after the first year of treatment): Commentary. Mult Scler 2013;19(8):1007-8.

s95. Zhang H, Schneider T, Wheeler-Kingshott CA, et al. NODDI: practical in vivo neurite orientation dispersion and density imaging of the human brain. Neuroimage 2012;61(4):1000-16.

s96. Whittall KP, MacKay AL, Graeb DA, et al. In vivo measurement of T2 distributions and water contents in normal human brain. Magn Reson Med 1997;37(1):34-43.

s97. Kamnitsas K, Ledig C, Newcombe VFJ, et al. Efficient multi-scale 3D CNN with fully connected CRF for accurate brain lesion segmentation. Med Image Anal 2017;36:61-78.

s98. Ledig C, Kamnitsas K, Koikkalainen J, et al. Regional brain morphometry in patients with traumatic brain injury based on acute- and chronic-phase magnetic resonance imaging. PLoS One 2017;12(11):e0188152.

s99. Kempton MJ, Ettinger U, Schmechtig A, et al. Effects of acute dehydration on brain morphology in healthy humans. Hum Brain Mapp 2009;30(1):291-8.

s100. Reuter M, Tisdall MD, Qureshi A, et al. Head motion during MRI acquisition reduces gray matter volume and thickness estimates. Neuroimage 2015;107:107-15.

s101. Jovicich J, Marizzoni M, Sala-Llonch R, et al. Brain morphometry reproducibility in multi-center 3T MRI studies: A comparison of cross-sectional and longitudinal segmentations. NeuroImage 2013;83:472-84.

s102. Konigs M, Beurskens EA, Snoep L, et al. Effects of Timing and Intensity of Neurorehabilitation on Functional Outcome After Traumatic Brain Injury: A Systematic Review and Meta-Analysis. Arch Phys Med Rehabil 2018;99(6):1149-59.e1. doi: 10.1016/j.apmr.2018.01.013

s103. Rafii MS, Aisen PS. Alzheimer’s Disease Clinical Trials: Moving Toward Successful Prevention. CNS Drugs 2019;33(2):99-106.

s104. Hickey GL, Grant SW, Dunning J, et al. Statistical primer: sample size and power calculations—why, when and how? European Journal of Cardio-Thoracic Surgery 2018;54(1):4-9.

s105. Hua X, Lee S, Yanovsky I, et al. Optimizing power to track brain degeneration in Alzheimer's disease and mild cognitive impairment with tensor-based morphometry: an ADNI study of 515 subjects. Neuroimage 2009;48(4):668-81.

s106. Tong KA, Ashwal S, Holshouser BA, et al. Diffuse axonal injury in children: clinical correlation with hemorrhagic lesions. Ann Neurol 2004;56(1):36-50. doi: 10.1002/ana.20123

s107. Thelin EP, Zeiler FA, Ercole A, et al. Serial Sampling of Serum Protein Biomarkers for Monitoring Human Traumatic Brain Injury Dynamics: A Systematic Review. Front Neurol 2017;8:300.

s108. Bogoslovsky T, Wilson D, Chen Y, et al. Increases of Plasma Levels of Glial Fibrillary Acidic Protein, Tau, and Amyloid beta up to 90 Days after Traumatic Brain Injury. J Neurotrauma 2017;34(1):66-73.
